# Molecular Characterization of the Recombinant Ig1 Axl Receptor Domain: An Intriguing Bait for Screening in Drug Discovery

**DOI:** 10.3390/molecules29020521

**Published:** 2024-01-20

**Authors:** Rossella Di Stasi, Lucia De Rosa, Guido Izzi, Luca Domenico D’Andrea

**Affiliations:** 1 Istituto di Biostrutture e Bioimmagini, CNR—Consiglio Nazionale delle Ricerche, Via Pietro Castellino 111, 80131 Napoli, Italy; lucia.derosa@cnr.it (L.D.R.); gu.izzi@studenti.unina.it (G.I.); 2 Istituto di Scienze e Tecnologie Chimiche “Giulio Natta”, CNR—Consiglio Nazionale delle Ricerche, Via Mario Bianco 9, 20131 Milano, Italy

**Keywords:** Axl receptor, recombinant Ig1 domain, protein refolding, drug discovery, circular dichroism, fluorescence spectroscopy, iodoacetamide, trypsin

## Abstract

Axl receptor tyrosine kinase and its ligand Gas6 regulate several biological processes and are involved in both the onset and progression of tumor malignancies and autoimmune diseases. Based on its key role in these settings, Axl is considered a promising target for the development of molecules with therapeutic and diagnostic purposes. In this paper, we describe the molecular characterization of the recombinant Ig1 domain of Axl (Ig1 Axl) and its biochemical properties. For the first time, an exhaustive spectroscopic characterization of the recombinant protein through circular dichroism and fluorescence studies is also reported, as well as a binding analysis to its natural ligand Gas6, paving the way for the use of recombinant Ig1 Axl as a bait in drug discovery screening procedures aimed at the identification of novel and specific binders targeting the Axl receptor.

## 1. Introduction

Axl is one of the three members of the TAM (tro-3, axl, and mer) receptor tyrosine kinase (RTK) family activated with the high-affinity ligands Gas6 (growth arrest-specific protein 6) and protein S (ProS) [1,2]. In particular, Axl and its specific ligand, Gas6, regulate a plethora of cellular processes, among which proliferation, survival, migration, angiogenesis, and innate immune responses participate in the development and progression of a range of malignancies and autoimmune disorders [3,4], as well as plays a fundamental role in viral entry mechanisms [5,6]. Axl receptor over-expression has been found in several cancer types and is often correlated with a patient’s poor prognosis and outcomes. In fact, Axl/Gas6 signaling triggers the epithelial-to-mesenchymal transition (EMT) phenomenon, playing a pivotal role in promoting metastases and improving drug resistance mechanisms [7,8,9]. Axl, along with the other TAM family members, shares the typical RTK architecture constituting an extracellular domain, a transmembrane region, and a conserved intracellular kinase domain. The extracellular portion is, in turn, structured into two immunoglobulin-like (Ig) repeats and two fibronectin type III (FNIII) repeats, recalling the organization of cell adhesion molecules, such as neural cell adhesion molecules (NCAMs), in which they are both important in cell-cell contacts [10]. Gas6 ligand binding to Axl promotes receptor dimerization, leading to the autophosphorylation of multiple tyrosine residues in the cytoplasmic domain and to the activation of downstream signaling pathways, including PI3K/AKT, MAPK/ERK, and STAT3 [1,11]. Previous studies have shown that the binding site of Gas6 to Axl involves the C-terminal LG1/LG2 domains of Gas6 [12,13]. The crystal structure resolved for the molecular complex formed by the Axl fragment spanning the two N-terminal Ig domains (Ig1/Ig2) and Gas6 LG1-LG2 domains provided additional insights, highlighting the two types of Gas6/Axl contacts leading to receptor activation. Only the Gas6 LG1 domain participates in Axl binding, whereas both the Ig1 and Ig2 repeats of Axl are involved in Gas6 recognition. The receptor-ligand major contact site is established between the β strand B of the Gas6 LG1 domain and the β strand D of the Axl Ig1 domain. At this major contact site, mutations in Axl Ig1-specific amino acid residues dramatically reduce Gas6 binding in vitro, while mutations in Axl or Gas6 at the minor contact site do not appreciably affect their high-affinity interaction [14]. Because of its pleiotropic role in tumor and immune diseases, viral infections, and drug resistance, the Axl receptor represents a promising therapeutic target [15]. Over the past years, different therapeutic agents have been developed, including numerous small molecules, anti-Axl monoclonal antibodies (mAbs), aptamers, and soluble receptors. They were not completely effective in blocking the Axl/Gas6 molecular axis, particularly in MET-amplified patients. However, clinical trials are currently in progress, and no drugs have been approved so far [16,17]. Thus, the challenge is to identify novel, specific, and active Axl targeting agents to be used as homing units in diagnostic applications or as therapeutic agents in single treatments or in combination with standard chemotherapy, overcoming the intrinsic limits of the molecules already developed and paving the way to increasingly personalized therapies. So far, a class of almost-unexplored molecules is represented by Axl-targeting peptides [18]. Peptide-based binders present several advantages over conventional drug molecules and could be used in a wide range of innovative diagnostic and therapeutic applications. In fact, peptide molecules can overcome the limitations of both small organic molecules and antibodies, i.e., off-target activity and a highly toxic profile for the former [19] and high production costs and poor tumor tissue penetration and delivery for the latter [20], instead showing favorable properties, such as high selectivity and safe pharmacological profiles [21,22]. In the present work, we describe an efficient and reliable methodology for the preparation of the recombinant human Ig1 Axl domain, reporting its biochemical and conformational characterization in a solution, as well as its binding analysis to Gas6. Gaining a correctly folded and functional recombinant Ig1 Axl protein is crucial for its use as bait in an interaction/screening assay aimed at finding new receptor binder molecules that can effectively target the Gas6/Axl axis.

## 2. Results

### 2.1. Expression and Purification of the His-Tagged Recombinant Ig1 Axl Protein

**Figure 1:**
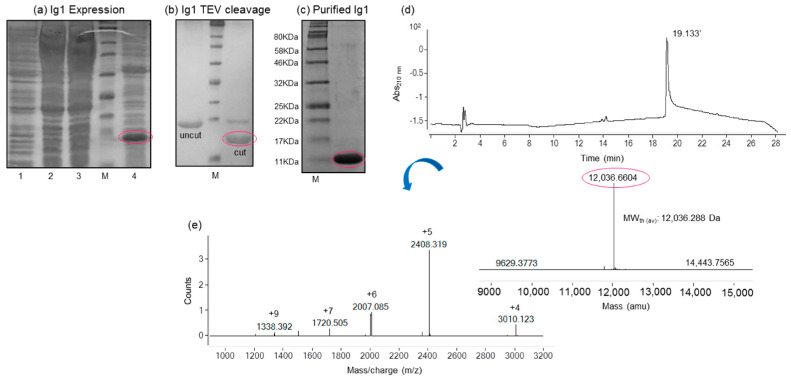
SDS-PAGE analysis of the (**a**) recombinant expression of the Ig1 Axl protein in the BL21-Gold(DE3)pLysS *E. coli* strain; (**b**) Ig1 Axl TEV cleavage; (**c**) recombinant Ig1 Axl concentrated in Amicon Ultra centrifugal filters ultracel after the size-exclusion chromatography purification step. Lane 1 of the gel (**a**) *E. coli* not induced crude extract in Tris-HCl 50 mM/NaCl 150 mM, pH = 7; Lanes 2 and 3: *E. coli*-induced crude extract supernatant in Tris-HCl 50 mM/NaCl 150 mM, pH = 7 (5 and 10 μL, respectively); Lane 4: *E. coli*-induced crude extract pellet in Tris-HCl 50 mM/NaCl 150 mM, pH = 7 (10 μL). In (**a**–**c**), Ig1 Axl is circled in the magenta ovals. M, pre-stained protein molecular weight marker (11–245 KDa). (**d**) the LC profile from the LC-MS ESI-ToF analysis of pure recombinant Ig1 Axl. The deconvoluted spectrum and the value of the average mass are reported in (**e**).

The primary coding amino acid sequence of the Ig1 Axl domain spans residues from 27 to 136 of the human Axl tyrosine kinase receptor, including 2 cysteine residues (Cys56, Cys117) involved in the formation of a conserved disulfide bridge (PDB_2C5D), typical of the immunoglobulin superfamily fold [23]. The gene corresponding to Ig1 Axl was amplified starting from the cDNA of the entire Axl receptor through two consecutive PCR steps. They were designed to introduce, upstream the domain sequence, a modified tobacco etch virus (TEV) protease recognition sequence. The fusion gene, once purified and digested, was cloned into the pET28b(+) vector downstream of a poly His affinity tag sequence, which was helpful for the first protein purification step via affinity chromatography. The His-tagged recombinant Ig1 Axl protein was insoluble and expressed in the BL21-Gold(DE3)pLysS *E. coli* strain (Figure 1a). Then, it was extracted from the inclusion bodies in a buffer containing 8 M urea and purified in batch via affinity chromatography on a Ni^+2^-NTA resin. Fractions containing the Ig1 Axl protein were eluted from the resin at high imidazole concentrations and pooled. The protein was then diluted dropwise in a buffer free of urea containing the thiol shuffling agents reduced glutathione (3 mM)/oxidized glutathione (0.3 mM) to facilitate disulfide bridge formation. The refolding procedure was allowed to proceed overnight (O/N) with stirring at 4 °C, resulting in a clear protein solution to which the TEV protease was added for the poly-histidine tag removal. The cleavage reaction proceeded O/N at 4 °C, with a proteolysis efficiency of over 90% (Figure 1b). The digested recombinant Ig1 Axl was finally purified to homogeneity with a single chromatography step using size-exclusion chromatography. The fractions corresponding to the purified protein were pooled and concentrated until a concentration of 0.30 mM was achieved (Figure 1c). Overall, a yield of 3 mg of pure recombinant Ig1 Axl per liter of *E. coli* culture was obtained. Subsequently, the pure protein was subjected to biochemical and conformational characterization in order to verify the correct fold.

### 2.2. Recombinant Ig1 Axl Protein Disulfide Bond Analysis

**Figure 2:**
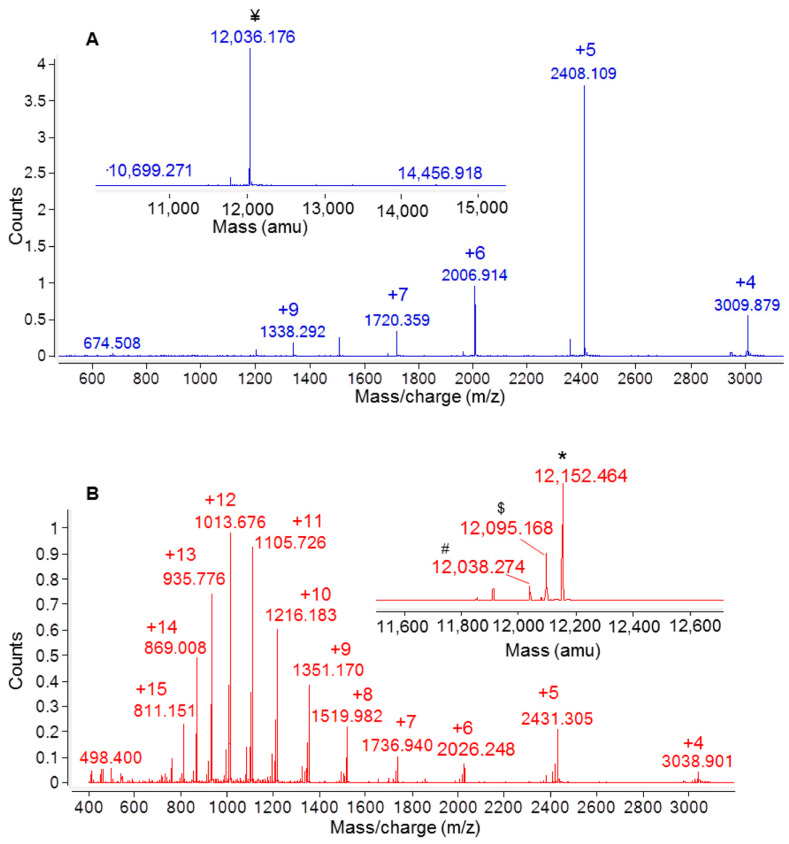
Analysis with ESI-ToF mass spectrometry of the recombinant Ig1 Axl domain after (**A**) incubation at 60 °C for 90 min and alkylation with iodoacetamide at RT for 30 min and (**B**) incubation with tris-carboxyethylphosphine (TCEP) at 60 °C for 90 min and alkylation with iodoacetamide at RT for 30 min. In the inset of panels, (**A**,**B**) report the deconvoluted mass spectra showing the experimental average mass values of the species. ^¥^ Ig1 Axl, MW_th_ (av): 12,036.288 Da; * dialkylated Ig1 Axl, MW_th_ (av): 12,152.388 Da; ^$^ monoalkylated MW_th_ (av): 12,095.338 Da; ^#^ reduced Ig1 Axl, MW_th_ (av): 12,038.288 Da.

**Figure 3:**
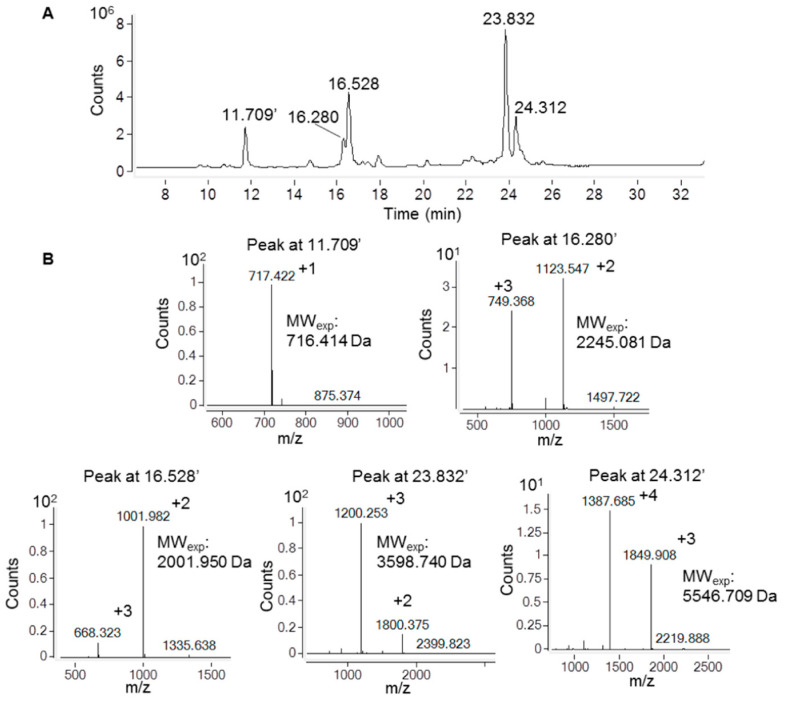
Analysis using LC-ESI-ToF mass spectrometry of the Ig1 Axl trypsin digestion mixture after 3 h of incubation with trypsin at 37 °C. (**A**) Chromatographic profile revealed by registering the total ionic current. (**B**) ESI-ToF spectra of the peptide fragments obtained.

**Figure 4:**
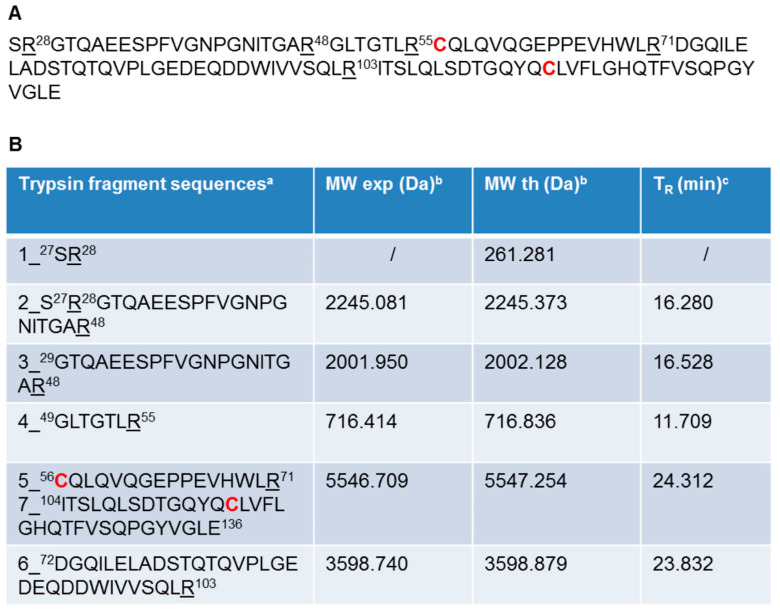
Ig1 Axl trypsin proteolytic digestion mixture analyzed with LC-MS after 3 h of incubation at 37 °C. (**A**) Ig1 Axl protein sequence in which the trypsin cleavage sites are underlined and the corresponding residue is numbered. The numbering refers to the human Axl receptor protein sequence annotated in the UniProt Database (entry P30530). The cysteine residues are in bold red. (**B**) List of trypsin-derived peptides of recombinant Ig1 Axl. ^a^ Amino acid sequences of the peptide fragments obtained from recombinant Ig1 Axl after trypsin digestion; ^b^ experimental (exp) and theoretical (th) molecular weight (MW) of the LC-MS-identified peptide fragments; ^c^ chromatographic retention time (T_R_) of the identified peptide fragments. The peptide fragments ^56^**C**–R^71^ and ^104^I–E^136^ reported in panel (**B**) correspond to a peptide fragment harboring the cysteine residues (^56^**C** and ^117^**C**) in the oxidized state joined with the disulfide bridge.

The oxidation state of cysteine residues involved in the formation of Ig1 Axl disulfide bond was evaluated by performing protein alkylation with iodoacetamide. An LC-MS analysis of the reactions kept in the dark for 30 min at room temperature (RT) demonstrated that the cysteine residue alkylation of recombinant Ig1 Axl only occurs after protein reduction with 20 mM TCEP. The LC-MS spectra of the alkylated and reduced/alkylated proteins are shown in Figure 2A and Figure 2B, respectively. The analysis revealed that the Ig1 Axl disulfide bond, after incubation at 60 °C for 90 min, remains intact, as the molecular mass of the heated alkylated protein (MW: 12,036.176, Figure 2A) does not change compared to that of the untreated protein (Figure 1d). Instead, when recombinant Ig1 Axl was reduced with TCEP 20 mM for 90 min at 60 °C and then alkylated with iodoacetamide 14 mM for 30 min at RT, the mass spectrum clearly shows the presence of the double alkylated protein as the main protein adduct (MW_th (av)_: 12,152.388 Da; MW_exp (av)_: 12,152.464 Da, Figure 2B). To further confirm the oxidation state of Cys^56^ and Cys^117^ in Ig1 Axl, the trypsin digestion of the protein was performed. The protease was added to recombinant Ig1 Axl in a molar ratio enzyme:Ig1 Axl of 1:100. The LC-MS spectrum of the digested sample was acquired after 3h of incubation at 37 °C, allowing the identification of five fragments covering the full protein sequence (100%) (Figure 3). A fragment eluted at a retention time of 24.312 min showed a mass peak of 5546.709 Da. This mass value is in agreement with that calculated for the fragments 5 C^56^–R^71^ and 7 I^104^–E^136^ (MW_th_: 5547.254 Da) derived from the theoretical trypsin digestion pattern, with Cys^56^ and Cys^117^ joined with the disulfide bond, as expected (Figure 4).

### 2.3. Recombinant Ig1 Axl-Gas6 Binding Analysis

**Figure 5:**
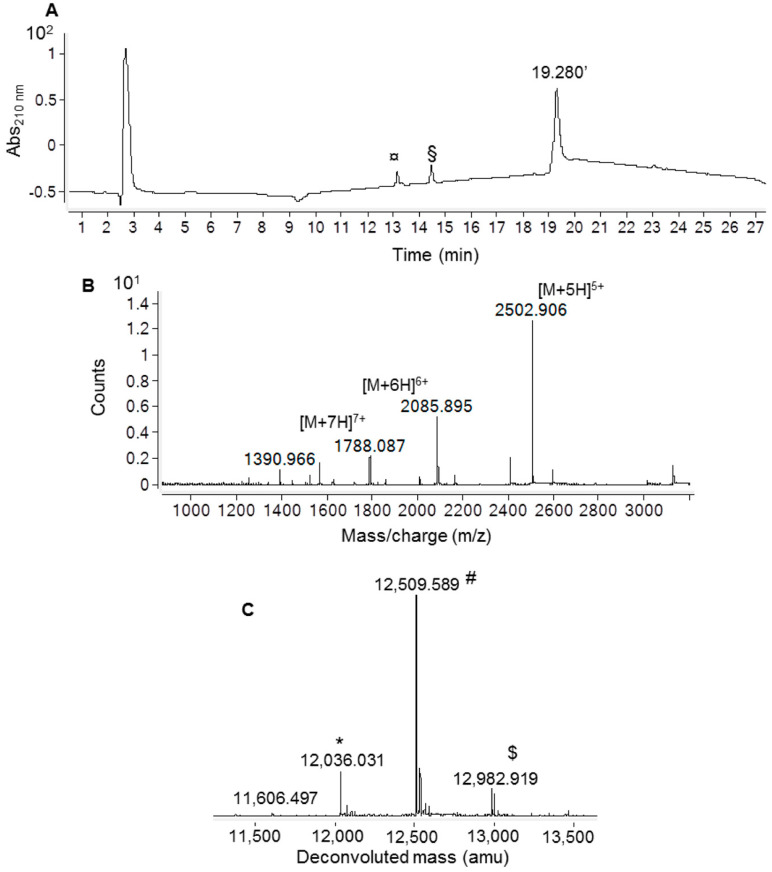
Analysis with LC-ESI-ToF mass spectrometry of the Ig1 Axl biotinylation reaction mixture after 1 h of incubation with NHS-dPEG^®^4-biotin at RT. (**A**) RP-HPLC chromatographic profile revealed by registering the absorbance at 210 nm. (**B**) ESI-ToF mass spectrum of the protein species eluted at 19.280 min. (**C**) Deconvoluted mass spectrum. * residual unlabeled Ig1 Axl (MW_th_: 12,036.288 Da); ^#^ Ig1 Axl labeled with dPEG^®^4-biotin (MW_th_: 12,509.868 Da); ^$^ trace amount of Ig1 Axl labeled with two dPEG^®^4-biotin units (MW_th_: 12,983.448 Da). The minor peaks eluted at 13.137 min and 14.457 min correspond to the reactive probe NHS-dPEG^®^4-biotin (§) and to the hydrolysis product of the probe dPEG^®^4-biotin (^¤^).

**Figure 6:**
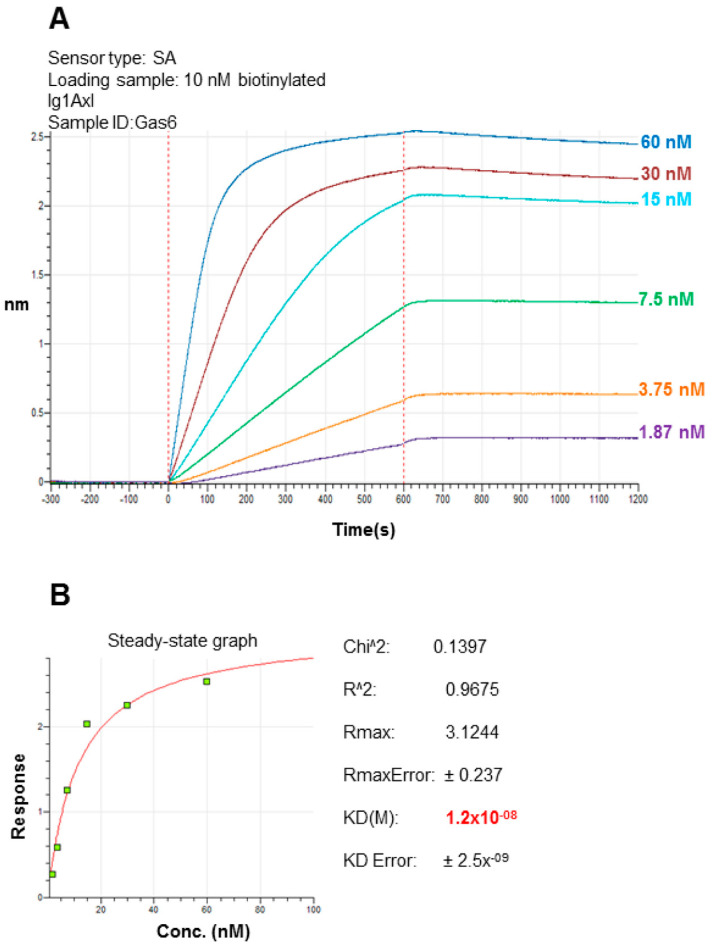
Binding analysis of recombinant Ig1 Axl to Gas6 LG1-LG2 with bio-layer interferometry. (**A**) Binding traces of biotinylated Ig1 Axl (10 nM) incubated with the indicated concentrations of Gas6 LG1-LG2. (**B**) Steady-state analysis derived by the fitting of the association and dissociation curves performed using the global algorithm and the 1:1 binding model.

The ability of recombinant Ig1 Axl to bind to Gas6 was evaluated by performing biolayer interferometry (BLI) experiments. To this aim, Ig1 Axl was biotinylated using an N-hydroxy succinimidyl ester derivative of the biotin (NHS-dPEG^®^4-biotin) as the reactive probe, which reacts in native conditions (mild temperatures and buffer solutions at a pH around neutrality) with protein amine groups, leading to the formation of an amide conjugate through an acylation reaction. The probe was expected to selectively react with the protein alpha-amino-terminal group [24], as recombinant Ig1 Axl does not bear any lysine residue in its amino acid sequence. The reaction was allowed to proceed for 1 h at RT. The probe excess was then removed, and the final product was analyzed via LC-MS. The main reaction product observed was the domain labeled with one unit of dPEG^®^4-biotin (MW_exp_: 12,509.589 Da; MW_th_: 12,509.868 Da), resulting in a net mass increase of 473,269 Da, as expected for protein mono-biotinylation (Figure 5). The biotinylated Ig1 Axl was tethered on the biosensor surface, dipping streptavidin (SA)-coated tips into a solution containing 10 nM of the biotinylated recombinant protein. To quench free streptavidin on the SA biosensor tip, 10 μg/mL biocytin, a biotin analog, was used. For the association step, we chose a concentration range of the analyte (consisting of Gas6 LG1-LG2 domains) from 60 nM to 1.87 nM, based on the dissociation constant (KD) value estimated for the Ig/Gas6 complex. The biotinylated Ig1 Axl-loaded SA tip was dipped in kinetics buffer 1X (KB 1X) and measured as a control to subtract from the experimental values before data processing. The non-specific interaction of the analyte with the biosensor was tested by dipping the SA biosensor tip in KB 1X diluted Gas6 LG1-LG2 solution at the maximum concentration value tested in the assay (60 nM). To this end, no biotinylated Ig1 Axl was added at the loading step. The sensorgrams obtained from the association and dissociation curves are reported in Figure 6A. They were fit using the global/1:1 binding model (Figure 6B), and a K_D_ of (1.2 ± 0.2) × 10^−8^ M was obtained, which is in agreement with the K_D_ value reported in the literature for the interaction of Axl Ig1-2 domains with Gas6 LG1-LG2 domains obtained using surface plasmon resonance (SPR) [14].

### 2.4. Spectroscopic Characterization of the Recombinant Ig1 Axl Protein

#### 2.4.1. Circular Dichroism (CD) Analysis

**Figure 7:**
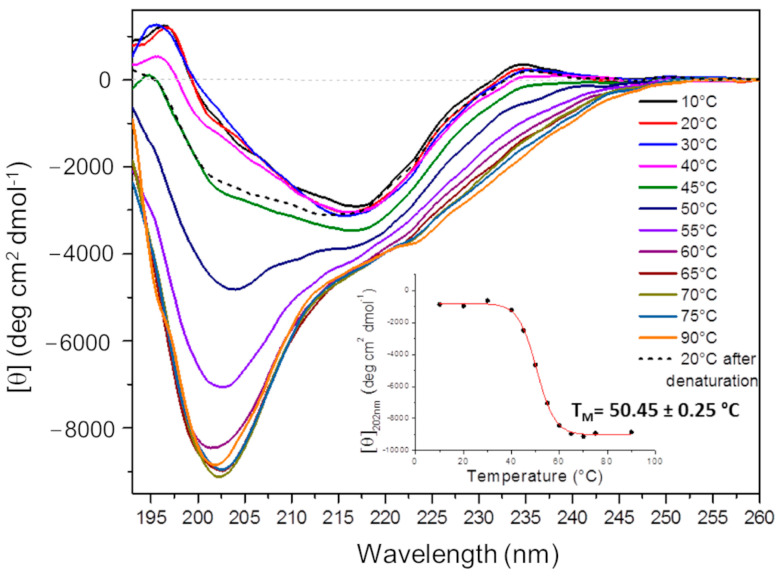
Recombinant Ig1 Axl thermal denaturation analysis using CD spectroscopy. The figure shows the superimposition of the CD spectra acquired in the temperature range of 10–90 °C. The data were collected over five averaged scans. The inset reports the thermal denaturation curve as the result of a non-linear regression analysis (R^2^ = 0.99) obtained by plotting the temperature dependence of the ellipticity values registered at 202 nm ([θ]_202_).

The Ig1 Axl secondary structure was studied using far-UV CD spectroscopy. Ig1 Axl is characterized by an all β fold, as revealed with x-ray crystallography [14]. The CD spectrum of Ig1 Axl was recorded at 20 °C with 12 μM of 10 mM phosphate buffer at pH 7. The spectrum acquired in the wavelength range of 193–260 nm displays a distinctive minimum at 217 nm and a positive band at ~195 nm, which are both hallmarks of β-sheet structures [25] (Figure 7, red curve). The thermal denaturation of the recombinant Ig1 Axl protein was performed by acquiring protein spectra at different temperatures between 10 and 90 °C (Figure 7). The superimposition of these spectra is reported in Figure 7, together with the spectrum of the Ig1 Axl domain acquired at 20 °C after denaturation at 90 °C. As the temperature increased, we observed an increase in the negative minimum around 200 nm and a progressive flattening of the band around 217 nm, which could suggest a possible contribution of both random coil and polyproline content in the denatured protein [26]. The inset of Figure 7 shows the sigmoidal curve obtained by plotting the ellipticity values recorded at 202 nm ([θ]_202_) as a function of temperature. The progressive increase in the ellipticity value at 202 nm as the temperature increases is in agreement with what is expected for a two-state (folded-unfolded) protein thermal denaturation process. The thermal denaturation midpoint (Tm) obtained for Ig1 Axl is 50.45 ± 0.25 °C, attesting to good thermal stability that, combined with the β-sheet content, suggests a correct fold of the recombinant Ig1 Axl protein. The CD spectra deconvolution was performed to calculate the percentage of each secondary structure present in the refolded Ig1 Axl protein. To this aim, we used the CDPro software with the protein dataset SPD48 (this was chosen as it also contains denatured proteins) and three algorithms (SELCON3, CDSSTR, and CONTINLL) [27,28,29], comparing the measurements acquired at 20 °C, 90 °C, and at 20 °C after denaturation (Table 1). The comparison between the spectra acquired at 20 °C before and after protein denaturation at 90 °C reveals an average β-sheet secondary structure content of approximately 37% and 27%, respectively (the values were obtained from the arithmetic mean of the β-sheet percentages calculated with the three algorithms), attesting to only a partially reversible denaturation process for Ig1 Axl, probably due to the onset of protein aggregation at high temperatures. A sensitive loss in the secondary structure from 20 °C to 90 °C was observed instead, as Ig1 Axl β-sheet content decreased until approximately 16% at 90 °C, and the unordered fraction increased to more than 60%.

#### 2.4.2. Steady-State Fluorescence Studies

**Figure 8:**
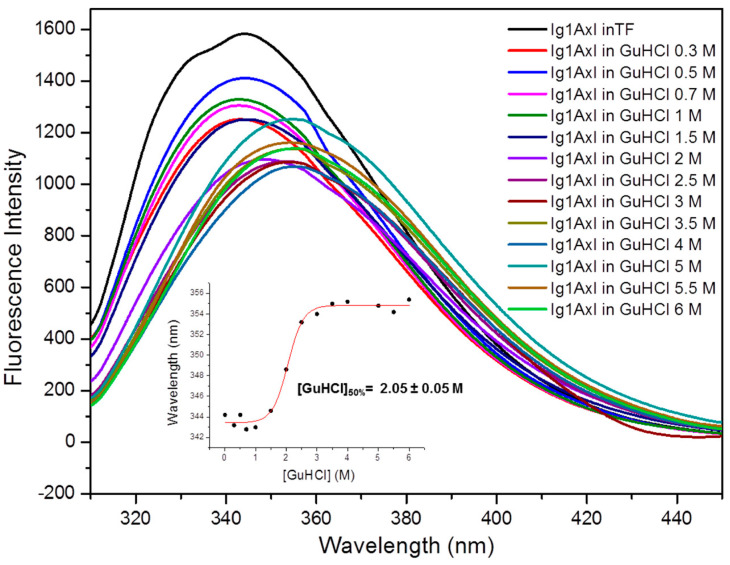
Ig1 Axl analysis created with steady-state fluorescence spectroscopy. The figure reports the superimposition of the Ig1 Axl emission spectrum recorded at 20 °C in 10 mM phosphate buffer (TF) at pH 7 (black curve) and the protein spectra acquired in the presence of increasing concentrations of GuHCl (colored curves from 0.3 to 6 M GuHCl). The inset shows the sigmoidal curve obtained by plotting the wavelength shift of the maximum fluorescence intensity as a function of GuHCl concentration, which represents the result of the non-linear regression analysis (R^2^ = 0.99).

We investigated the conformational properties of the recombinant Ig1 Axl protein using steady-state fluorescence spectroscopy because of the presence of its amino acid sequence, which includes two tryptophan residues (Trp^69^ and Trp^96^). In order to minimize the emission of the other aromatic residues of Ig1 Axl, we set the excitation wavelength to 295 nm. The Ig1 Axl fluorescence emission spectrum acquired at RT with 0.2 μM of a 10 mM phosphate buffer at pH 7 shows a curve whose absorption maximum is centered near the value of 344 nm (black curve in Figure 8), suggesting that the Trp residues in the recombinant Ig1 Axl protein could be partially exposed to the solvent. Titration with increasing concentrations of guanidinium hydrochloride (GuHCl) resulted in a progressive shift in the wavelength at which the maximum fluorescence absorption occurred, indicating that the Trp residues do not experience the same chemical environment in the unfolded protein compared to the folded protein (Figure 8). By plotting the maximum fluorescence absorption value of each curve as a function of GuHCl concentrations, we obtained a sigmoidal denaturation curve, which represents a typical unfolding behavior for a globular protein occurring through a two-state mechanism. The value of GuHCl concentration at which half of the protein molecules are unfolded ([GuHCl]_50%_) was 2.05 ± 0.05 M, indicating good chemical stability for Ig1 Axl. Data showing the red shift of the maximum fluorescence intensity for each curve are reported in the inset of Figure 8.

## 3. Discussion

In recent decades, the Axl receptor, due to its pleiotropic role in a wide range of physio-pathological processes, has emerged as a novel biomarker to target in the treatment of disorders. This is derived from its aberrant signaling, leading to pathological settings, such as cancer and autoimmune diseases, among others [3,4,30]. For this reason, in recent years, medical research, especially tumor physiology research, has focused on the study of the Axl/Gas6 molecular axis, resulting in the development of different classes of therapeutic agents, from kinase inhibitors to anti-receptor antibodies and aptamers, even if no drug has been FDA-approved to date [16,17]. The binding of Gas6 to the extracellular portion of Axl triggers receptor dimerization, trans-phosphorylation, and activation. The extracellular portion of Axl involved in Gas6 binding is composed of two Ig-like domains. Mutagenesis studies have shown that the Axl/Gas6 high-affinity interaction is accomplished between the first Ig-like domain of the Axl receptor (Ig1) and the LG1 domain of soluble Gas6. Although the three-dimensional X-ray structure of the complex Ig1-2 Axl/Gas6 LG1-LG2 domains was resolved by Sasaki and coworkers [14], no spectroscopic and biochemical characterization has been described so far for the Ig1 domain. This could provide useful insights to verify Ig1 Axl protein properties and to set up appropriate binding experiments aimed at the identification of new specific binders for the Axl receptor. In particular, the development of a reliable experimental procedure for the obtainment of the properly folded recombinant Ig1 Axl protein is mandatory for its use as a bait in drug discovery screening experiments. The nucleotide sequence corresponding to the Ig1 Axl domain was successfully cloned and expressed in *E. coli*. A refolding protocol was successfully set up by diluting the protein domain solubilized in 8 M urea drop by drop into a solution free of the denaturant agent. The refolding procedure was a pivotal step and allowed us to move on in a relatively short time with low consumption of urea. After tag removal and purification via size-exclusion chromatography, we obtained the homogeneous protein for molecular characterization studies. Previously, Kariolis et al. reported the recombinant expression of the Ig1 Axl protein in *P. pastoris* [31]. Our experimental procedure involves, as in their case, two consecutive purification steps. Unfortunately, they do not report any reference to the expression levels and the final yield of pure protein obtained in the yeast host, so we cannot compare the efficiency of the two procedures. Once identified using LC-MS analysis, the correct fold of recombinant Ig1 Axl and its thermal stability were assessed using CD spectroscopy experiments. The analyses confirmed the Ig1 Axl β-sheet content expected for an Ig-like domain, which was in good agreement with the secondary structure content of Ig1 Axl derived from the crystal structure reported in the literature [14]. The recombinant Ig1 Axl protein presents a Tm of approximately 50 °C, which is in agreement with what is observed for stable and folded Ig-like domains. The denaturation process is almost completely reversible, as mild protein aggregation at high temperatures could occur. Structural data from Sasaki and coworkers showed that the two cysteine residues present in the Ig1 Axl amino acid sequence are engaged in a disulfide bridge. In order to verify the oxidation state of the cysteinyl sulfhydryl groups in the recombinant Ig1 Axl protein, we performed iodoacetamide alkylation and trypsin digestion experiments. Both analyses were examined using LC-MS and confirmed that Cys^56^ and Cys^117^ are involved in the formation of the expected disulfide bond, which remains intact and stable even when incubating the protein at 60 °C for 90 min. The activity of recombinant Ig1 Axl was assessed by evaluating Gas6/Ig1 Axl binding via bio-layer interferometry experiments. Fitting the association and dissociation curves resulted in an affinity value for the interaction between Gas6 LG1-LG2 and the recombinant Ig1 Axl protein in the low nanomolar range. This finding is in agreement with the SPR data reported in the literature on the interaction of Axl Ig1–2/Gas6 LG1-LG2 [14]. This binding data confirmed that the Ig1-Axl domain is sufficient for the interaction with Gas6 LG1-LG2. Furthermore, this result demonstrates the robustness of the procedure developed to refold the Ig1 Axl domain from the inclusion bodies and provides a valid tool to use in screening study design. Ig1 Axl presents two tryptophan residues in its amino acid sequence, which allowed us to follow protein unfolding via fluorescence spectroscopy, resulting in useful insights about its chemical stability. The protein fluorescence emission spectrum showed a maximum value of approximately 344 nm, which is typical of a Trp exposed to a polar solvent. Chemical denaturation performed in the presence of increasing concentrations of GuHCl resulted in a shift towards higher wavelengths (the red shift), indicative of the variation in the chemical environment experienced by the tryptophan(s) residues in the denatured protein. A sigmoidal denaturation curve was obtained by plotting the maximum fluorescence absorption value of each curve as a function of the denaturant agent concentration. This is what is expected for the two-state unfolding transition of a globular protein domain. The half transition value obtained from the denaturation curve ([GuHCl]_50%_) agrees with those reported for other similar Ig-like domains [32,33] and is indicative of good chemical stability of recombinant Ig1 Axl.

## 4. Materials and Methods

### 4.1. Reagents, Strains, and Plasmids

The reagents for the preparation of *Escherichia coli* (*E. coli*) growth medium (yeast extract and bacto tryptone), as well as the reagents for the buffer solutions (including oxidized and reduced glutathione, ethylenediaminetetraacetic acid ((EDTA)), were obtained from Neofroxx (Einhausen, Germany). The reagents for the agarose and polyacrylamide gels electrophoresis were supplied from Deltek (Napoli, Italy) (acrylamide, SDS, Tris, glycine), Sigma Aldrich (Milano, Italy) (tetramethylethylenediamine(TEMED)), and Euroclone (Milano, Italy) (ammonium persulfate (APS)). The molecular weight markers were from Neofroxx for the proteins and from New England Biolabs (NEB, Ipswich, MA, USA) for the DNA. The restriction and modification enzymes (calf intestine phosphatase and T4 DNA ligase) were supplied from NEB, Taq DNA polymerase (5 U/μL) was supplied from Euroclone, while Pfu Turbo polymerase (2.5 U/μL) was supplied from Agilent Technologies (Santa Clara, CA, USA). The pET28b(+) *E. coli* expression plasmid was supplied from Novagen (Madison, WI, USA). The BL21-Gold(DE3)pLysS *E. coli* competent cells for recombinant Ig1 Axl expression were from Agilent Technologies, while the DH5α *E. coli* competent cells for the propagation of the recombinant plasmids were from Invitrogen. The DNA purification kits and Ni^2+^-NTA resin were purchased from Qiagen (Venlo, The Netherlands) while the synthesis of the oligonucleotides and the sequencing service were commissioned at Eurofins Genomics. GuHCl 8 M was employed for the spectroscopic denaturation experiments, Tris(2-carboxyethyl)phosphine hydrochloride (TCEP-HCl) was used for the thiol group reduction, iodoacetamide was used for the cysteine alkylation of Ig1 Axl, and the trypsin for the protein domain enzymatic digestion were all reagents from Sigma Aldrich. The streptavidin (SA) biosensors and Kinetic Buffer 10X for the biolayer interferometry (BLI) measurements were purchased from Sartorius. The His-tagged Gas6 LG1-LG2 protein for the BLI assays was supplied from Acro Biosystems (Newark, DE, USA).

### 4.2. Ig1 Axl Protein Expression, Refolding, and Purification

The DNA sequence corresponding to the Ig1 domain of the Axl receptor (amino acid residues 27–136 of human Axl, UniProt Database entry N. P30530) was amplified in two consecutive PCR steps in order to add a modified TEV protease cleavage site at the 5′-terminus of the gene, resulting in a serine residue as the first amino acid of Ig1 Axl protein domain. The first gene amplification reaction was performed using the Axl/pCMVneo vector as a template, which contained the cDNA of the entire gene, kindly provided by Dr. Axel Ullrich (Max Planck Institute of Biochemistry, Martinsried-Germany). For the first PCR step of Ig1 Axl, we used the following primers:

N-SerIg1Fw_ 5′-AAC CTG TAT TTT CAG AGT AGG GGC ACG CAG GCT-3′

Ig1Rv_ 5′GTG CTC GAG CTA TCA CTC CAG CCC AAC ATA GCC-3′

Where the sequence encoding the partially modified TEV site (with AGT = Ser in place of the first amino acid of the Ig1 domain, CCC = Pro) is in green, the *Xho*I restriction site is in red, and the stop codons are in bold. For the second PCR step of the Ig1 domain, we used the same reverse primer of the above PCR step and a new forward primer:

TEVSerIg1Fw_ 5′ G GAA TTC CAT ATG GAA AAC CTG TAT TTT CAG AGT-3′.

Where the *Nde*I restriction site is in blue and the completely modified TEV site is in green.

For both Ig1 Axl amplification steps, we used 50 ng of DNA template (Axl/pCMVneo vector DNA and purified first Ig1 PCR product), 5 U of Pfu Turbo DNA polymerase (2.5 U/μL), 30 pmol of each primer, and 0.25 mM of each dNTP in a final PCR volume of 100 μL. The annealing temperature was 45 °C in both cases. The final product of the Ig1 Axl protein gene amplification, visualized via fluorescence as a single product (376 bp), was then purified using a Qiagen PCR purification kit and digested with 4 U/µg of *Nde*I (10 U μL^−1^) and *Xho*I (20 U μL^−1^) restriction enzymes (NEB). The *Nde*I/*Xho*I gene fragment was purified and ligated according to a molar ratio of vector: inset of 1:5 into the *Nde*I/*Xho*I sites of the pET28b(+) expression vector polylinker for 3 h at room temperature (RT). The reaction mix was set up using 100 ng of *Nde*I–*Xho*I pET28b in a final volume of 10 μL containing 20 U of the T4 DNA ligase (400 U/μL). This cloning strategy allowed for the recombinant expression of human Ig1 Axl with an additional 6x-His tag at the N-terminus of the protein domain downstream of the TEV recognition site sequence. The identity of the insert in the resulting recombinant plasmid was confirmed using DNA sequencing commissioned to Eurofins Genomics. The Ig1 Axl domain was expressed in the BL21-Gold(DE3)pLysS *E. coli* strain transformed with 100 ng of the recombinant Ig1 Axl/pET28b expression vector. The cells were grown overnight (O/N) with shaking at 37 °C in the presence of kanamycin (50 μg/mL) and chloramphenicol (33 μg/mL) antibiotics. The cells were then inoculated in fresh medium containing antibiotics for the preparative culture. Once the exponential bacterial growth phase (0.7/0.8 OD_600nm_) was reached, cell culture was induced by adding 1 mM IPTG, and the cells were allowed to incubate at 37 °C O/N (16 h). The pellet derived from 1 L of culture was resuspended in 25 mL of a lysis buffer containing 50 mM Tris-HCl and 150 mM NaCl pH 7 and sonicated for 10 min by using a Misonix Sonicator 3000 apparatus equipped with a macro tip probe applying an impulse output of 0.5/1 (=20/24 Watt). The lysate was centrifuged at 18,000 rpm for 30 min at 4 °C (a Beckman centrifuge equipped with a JA25.50 rotor), and the supernatant and the pellet samples were analyzed using SDS-PAGE on a 15% polyacrylamide gel. His-tagged Ig1 Axl was extracted from the inclusion bodies by means of the lysis buffer. Then, 10 mM imidazole and 8 M urea were added. Then, the protein was loaded in batch on a Ni^2+^-NTA resin (with constant stirring for 40 min at RT) and eluted with a high concentration of imidazole (from 250 to 300 mM). The fractions eluted from the resin were collected and analyzed via SDS-PAGE. Those containing the protein domain were pooled, and the concentrations of the proteins in the solution were determined according to Bradford’s method, adding the Coomassie Brilliant (Bio-Rad, Tokyo, Japan) reagent to the samples and monitoring their absorbance at 595 nm. A solution of 1 μg/μL of bovine serum albumin (BSA, Washington, DC, USA) was used as the standard. Protein refolding was performed following the same procedure as described by the authors [33]. Briefly, Ig1 Axl was diluted drop by drop at a final concentration of 0.01 mg/mL into a refolding buffer containing 50 mM Tris-HCl, 150 mM NaCl, and 3 mM reduced glutathione/0.3 mM oxidized glutathione at pH 7. The refolding was carried out at 4 °C O/N with stirring. Then, the TEV protease (expressed and purified in our lab) was added to the refolded protein for His-tag removal. A hydrolysis reaction was performed using a molar ratio of 1:35 (protease:substrate) in the presence of EDTA 0.5 mM, O/N at 4 °C. The cleaved protein was concentrated using the Amicon Ultra centrifugal filters ultracel with a molecular weight cut off of 10 KDa (Millipore, Burlington, MA, USA) and purified to homogeneity via size-exclusion chromatography on a Superdex 75_10/30_ column (GE Healthcare, Chicago, IL, USA) equilibrated in 50 mM Tris-HCl and 150 mM NaCl at pH 7. Finally, it was concentrated again until 0.30 mM. The pure Ig1 Axl protein concentration was determined by measuring the protein absorbance at 280 nm on a Nanodrop microvolume UV-Vis spectrophotometer (Thermo scientific, Waltham, MA, USA) using a molar extinction coefficient of 14,105 cm^−1^ M^−1^ [https://web.expasy.org/protparam/] (accessed on 7 October 2023). The identity of the protein before and after TEV cleavage was assessed via an LC-MS analysis performed on an Agilent 1200 Infinity Series apparatus (Agilent Technologies) equipped with an ESI source and a ToF detector using a Jupiter C4 column (150 × 2.0 mm, 5 μm, 300 Å) (Phenomenex, Torrance, CA, USA) applying a method with a flow rate of 0.2 mL min^−1^ and a linear gradient of CH_3_CN (0.05% TFA) in H_2_O (0.05% TFA) from 5% to 70% (*v*/*v*) for 20 min.

### 4.3. Ig1 Axl Iodoacetamide Alkylation and Trypsin Digestion

In order to estimate the oxidation state of its two cysteine residues, recombinant Ig1 Axl was diluted up to 10.5 μM in 100 mM ammonium bicarbonate (AMBIC, Sigma Aldrich, Milano, Italy) buffer with a pH of 8.0 and incubated for 90 min in a block-heater set at 60 °C. Iodoacetamide (Sigma Aldrich, Milano, Italy) was dissolved at a concentration of 300 mM in the same buffer and added to the heat-treated protein domain at a final concentration of 14 mM. The reaction was allowed to proceed for 30 min at RT in the dark with gentle agitation. As a positive control, the protein domain was first reduced with 20 mM TCEP for 90 min at 60 °C and then alkylated using iodoacetamide at RT for 30 min in the dark. The identity of both the alkylated and reduced/alkylated proteins was assessed using LC-MS, as described in Section 4.2. The trypsin digestion reaction was performed with 200 μL of a 10.5 μM Ig1 Axl solution in 50 mM Tris-HCl at pH 7 containing 20 mM CaCl_2_ using a molar ratio of trypsin:Ig1 Axl of 1:100. The reaction was incubated at 37 °C and analyzed after 3 h with LC-MS.

### 4.4. In Vitro Ig1 Axl/Gas6 Binding Assay using Biolayer Interferometry (BLI)

The validation of the Ig1 Axl refolding procedure was carried out by evaluating the binding of the recombinant Ig1 Axl protein to the Gas6 LG1-LG2 domains. The measurements were performed on the Sartorius Octet R8 platform using streptavidin (SA)-coated biosensor tips. The chamber temperature of the instrument was kept constant at 30 °C. The biosensors were hydrated in a Greiner black 96-well plate containing 200 μL of kinetics buffer 1X (KB 1X = PBS 1X with 0.01% BSA and 0.002% Tween-20) for 10 min with a plate agitation speed of 1000 rpm. After the loading scouting procedure, we chose the concentrations of both the ligand and analyte to test in the assay; the SA biosensors were dipped into 200 μL of biotinylated Ig1 Axl diluted up to 10 nM in KB 1X for ligand immobilization. To this aim, the Ig1 Axl domain was first buffer-exchanged into PBS 1X by the use of PD Spintrap™ G-25 Cytiva columns supplied from Merck. Then, the protein domain was biotinylated in the presence of a 5-fold molar excess of NHS-dPEG^®^-4-biotin (Merck, Darmstadt, Germany). The biotinylated Ig1 Axl was purified on a PD Spintrap column and analyzed using LC-MS. Once loaded with biotinylated Ig1 Axl, the tips were then moved into the KB 1X buffer for a baseline step (300 s) followed by the quenching step (600 s) in a solution containing biocytin (Merck) diluted up to 10 μg/mL in KB 1X. After an additional baseline step of 300 s, the tips were moved into solutions containing 1:2 serial dilution concentrations (60, 30, 15, 7.5, 3.75, and 1.875 nM) of Gas6 LG1-LG2 in KB 1X to obtain the association curves. Following the 600 s of the association step, the tips were moved back into the KB 1X buffer to obtain the dissociation curves (600 s). The empty SA biosensor tips dipped in His-tagged Gas6 LG1-LG2 at 60 nM, and the SA biosensor tips loaded with Ig1 Axl ligand dipped in only KB 1X buffer were used as the references (the reference biosensor and reference well, respectively) for background subtraction. The association and dissociation curves were fitted using Octet Analysis Studio 12.1 software, using the global algorithm and the 1:1 binding model to calculate the dissociation constant (K_D_) for the recombinant Ig1 Axl/Gas6 LG1-LG2 interaction.

### 4.5. Recombinant Ig1 Axl Protein Circular Dichroism (CD) Analysis

In order to estimate the secondary structure content of the recombinant Ig1 Axl domain, far-UV circular dichroism (CD) experiments were performed. The spectra were acquired on a Jasco J-1500 spectropolarimeter equipped with a PTC-510 Peltier (Jasco, Tokyo, Japan) temperature controller using a 0.1 cm quartz cell (Hellma, Müllheim, Germany). The first Ig1 Axl spectrum was recorded at 20 °C in phosphate buffer 10 mM at pH 7 at a protein concentration of 12 μM. The spectra of the protein domain at the same concentration but at different temperature values from 10 °C to 90 °C were then collected in order to study the behavior as the temperature increased. All experiments were performed in the wavelength interval between 193 and 260 nm, setting a data pitch of 0.2 nm, 10 nm (or 50 nm for the spectra in the T range 10 °C–90 °C)/min scan speed, 1.0 nm bandwidth, and a 4 s response as the parameters. The data were collected over three/five averaged scans and expressed as mean residue ellipticity [Ɵ] after subtracting the buffer contribution using the Spectra Manager software, Version 2.15.01. The data analysis for all the acquired experiments was carried out using the software Origin Pro (version 8.5), using a non-linear regression fit as described by Greenfield [34] and a sigmoidal behavior. A comparison between the CD spectra deconvolution of Ig1 Axl at 20 °C, 90 °C, and 20 °C after thermal denaturation was performed using the CDPro software, and the protein reference was set to SPD48. The secondary structure content values were obtained as an average of the three algorithms (SELCON3, CDSSTR, and CONTINLL) used for the prediction analysis.

### 4.6. Fluorescence Spectroscopy Analysis of the Recombinant Ig1 Axl Protein

The Ig1 Axl fluorescence experiments were performed on a Jasco FP-8350 spectrofluorometer equipped with an ETC-115 Peltier (Jasco). The protein spectra were recorded at 20 °C in a 1.0 cm quartz cell (Hellma) using a protein sample prepared by diluting recombinant Ig1 Axl up to 0.2 μM in phosphate buffer 10 mM at pH 7. A denaturation analysis was performed in the same buffer containing increasing concentrations (0.3 M; 0.5 M; 0.7 M; 1 M; 1.5 M; 2 M; 2.5 M; 3 M; 3.5 M; 4 M; 5 M; 5.5 M; 6 M) of guanidine hydrochloride (GuHCl, Sigma-Aldrich), which was diluted from an 8 M stock solution. After the acquisition of the Ig1 Axl spectrum in the potassium phosphate buffer 10 mM at pH 7, the protein samples in the presence of increasing GuHCl concentrations were left to equilibrate for 40 min at RT. All the experiments were carried out as three averaged scans, setting a scan speed of 200 nm/min, exciting the samples at 295 nm, and recording the emission spectra in the range of 300–500 nm. The excitation and the emission slit widths were set at 5 nm. The denaturation curve was obtained, which reported the values of the maximum fluorescence intensities for each curve registered as a function of GuHCl molar concentration. The data were analyzed using the software Origin Pro (version 8.5) with a non-linear regression fit as reported for the CD experiments.

## 5. Conclusions

In conclusion, in this work, we describe the procedure for the recombinant expression of the Ig1 Axl protein in *E. coli* and a robust protocol for the obtainment of a folded and functional protein, which was then carefully investigated through biochemical, spectroscopic, and binding techniques that validated its correct fold and activity. We provided the CD signature of the recombinant Ig1 Axl protein and its secondary structure content in a solution. Furthermore, its thermal and chemical stability were also described. The ability to obtain such a protein allows for its use as a bait in drug discovery screening assays aimed at identifying novel binders able to target the Axl receptor.

## Figures and Tables

**Table 1:** Predictions of recombinant Ig1 Axl protein secondary structure content using CDPro software with the SPD48 protein dataset and three algorithms: Selcon3, Continll, and CDSSTR. In the table, the values (%) of the secondary structure content corresponding to the deconvolution of the Ig1 Axl CD spectra registered at 20 °C, 90 °C, and 20 °C after thermal denaturation (*) are reported.

Algorithm Used	% Helix	% Sheet	% Turn	% Unordered
Selcon3, 20 °C	10.5	36	16	37.5
Continll, 20 °C	3.6	37.2	20.9	38.3
CDSSTR, 20 °C	3.5	38.3	19.9	38.3
Selcon3, 90 °C	10.2	19.6	13.6	56.6
Continll, 90 °C	7.5	15.8	12.8	63.9
CDSSTR, 90 °C	7.7	13.9	11.4	66
Selcon3, 20 °C *	3.1	28.1	12.1	56.7
Continll, 20 °C *	6.6	28.2	19.2	46
CDSSTR, 20 °C *	14	25.9	18.5	41.6

## Data Availability

The data presented in this study are available within the present manuscript.

## References

[1] Tsou W.I., Nguyen K.Q., Calarese D.A., Garforth S.J., Antes A.L., Smirnov S.V., Almo S.C., Birge R.B., Kotenko S.V. (2014). Receptor tyrosine kinases, TYRO3, AXL, and MER, demonstrate distinct patterns and complex regulation of ligand-induced activation. J. Biol. Chem..

[2] Lew E.D., Oh J., Burrola P.G., Lax I., Zagorska A., Traves P.G., Schlessinger J., Lemke G. (2014). Differential TAM receptor-ligand-phospholipid interactions delimit differential TAM bioactivities. eLife.

[3] Wium M., Paccez J.D., Zerbini L.F. (2018). The Dual Role of TAM Receptors in Autoimmune Diseases and Cancer: An Overview. Cells.

[4] Bellan M., Pirisi M., Sainaghi P.P. (2016). The Gas6/TAM System and Multiple Sclerosis. Int. J. Mol. Sci..

[5] Meertens L., Labeau A., Dejarnac O., Cipriani S., Sinigaglia L., Bonnet-Madin L., Le Charpentier T., Hafirassou M.L., Zamborlini A., Cao-Lormeau V.M. (2017). Axl Mediates ZIKA Virus Entry in Human Glial Cells and Modulates Innate Immune Responses. Cell Rep..

[6] Shimojima M., Ikeda Y., Kawaoka Y. (2007). The mechanism of Axl-mediated Ebola virus infection. J. Infect. Dis..

[7] Nakano T., Tani M., Ishibashi Y., Kimura K., Park Y.B., Imaizumi N., Tsuda H., Aoyagi K., Sasaki H., Ohwada S. (2003). Biological properties and gene expression associated with metastatic potential of human osteosarcoma. Clin. Exp. Metastasis.

[8] Graham D.K., DeRyckere D., Davies K.D., Earp H.S. (2014). The TAM family: Phosphatidylserine sensing receptor tyrosine kinases gone awry in cancer. Nat. Rev. Cancer.

[9] Gjerdrum C., Tiron C., Hoiby T., Stefansson I., Haugen H., Sandal T., Collett K., Li S., McCormack E., Gjertsen B.T. (2010). Axl is an essential epithelial-to-mesenchymal transition-induced regulator of breast cancer metastasis and patient survival. Proc. Natl. Acad. Sci. USA.

[10] Yamagata M., Sanes J.R., Weiner J.A. (2003). Synaptic adhesion molecules. Curr. Opin. Cell Biol..

[11] Schlessinger J. (2000). Cell signaling by receptor tyrosine kinases. Cell.

[12] Mark M.R., Chen J., Hammonds R.G., Sadick M., Godowsk P.J. (1996). Characterization of Gas6, a member of the superfamily of G domain-containing proteins, as a ligand for Rse and Axl. J. Biol. Chem..

[13] Tanabe K., Nagata K., Ohashi K., Nakano T., Arita H., Mizuno K. (1997). Roles of gamma-carboxylation and a sex hormone-binding globulin-like domain in receptor-binding and in biological activities of Gas6. FEBS Lett..

[14] Sasaki T., Knyazev P.G., Clout N.J., Cheburkin Y., Gohring W., Ullrich A., Timpl R., Hohenester E. (2006). Structural basis for Gas6-Axl signalling. EMBO J..

[15] Di Stasi R., De Rosa L., D’Andrea L.D. (2020). Therapeutic aspects of the Axl/Gas6 molecular system. Drug Discov. Today.

[16] Zhu C., Wei Y., Wei X. (2019). AXL receptor tyrosine kinase as a promising anti-cancer approach: Functions, molecular mechanisms and clinical applications. Mol. Cancer.

[17] Bhalla S., Gerber D.E. (2023). AXL Inhibitors: Status of Clinical Development. Curr. Oncol. Rep..

[18] Kim J.H., Koh B., Ahn D.G., Lee S.J., Park T.J., Park J.P. (2021). A screening study of high affinity peptide as molecular binder for AXL, tyrosine kinase receptor involving in Zika virus entry. Bioelectrochemistry.

[19] Roskoski R. (2020). Properties of FDA-approved small molecule protein kinase inhibitors: A 2020 update. Pharmacol. Res..

[20] Cruz E., Kayser V. (2019). Monoclonal antibody therapy of solid tumors: Clinical limitations and novel strategies to enhance treatment efficacy. Biologics.

[21] Henninot A., Collins J.C., Nuss J.M. (2018). The Current State of Peptide Drug Discovery: Back to the Future?. J. Med. Chem..

[22] Wang L., Wang N., Zhang W., Cheng X., Yan Z., Shao G., Wang X., Wang R., Fu C. (2022). Therapeutic peptides: Current applications and future directions. Signal Transduct. Target. Ther..

[23] Zaccai N., Jones E.Y., Bradshaw R.A., Dennis E.A. (2003). Ig-Superfold and its Variable Uses in Molecular Recognition. Handbook of Cell Signaling.

[24] De Rosa L., Di Stasi R., Romanelli A., D’Andrea L.D. (2021). Exploiting Protein N-Terminus for Site-Specific Bioconjugation. Molecules.

[25] Greenfield N.J. (2006). Using circular dichroism spectra to estimate protein secondary structure. Nat. Protoc..

[26] Rucker A.L., Creamer T.P. (2002). Polyproline II helical structure in protein unfolded states: Lysine peptides revisited. Protein Sci..

[27] Provencher S.W., Glockner J. (1981). Estimation of globular protein secondary structure from circular dichroism. Biochemistry.

[28] Manavalan P., Johnson W.C. (1987). Variable selection method improves the prediction of protein secondary structure from circular dichroism spectra. Anal. Biochem..

[29] Sreerama N., Woody R.W. (2000). Estimation of protein secondary structure from circular dichroism spectra: Comparison of CONTIN, SELCON, and CDSSTR methods with an expanded reference set. Anal. Biochem..

[30] Gay C.M., Balaji K., Byers L.A. (2017). Giving AXL the axe: Targeting AXL in human malignancy. Br. J. Cancer.

[31] Kariolis M.S., Miao Y.R., Jones D.S., Kapur S., Mathews I.I., Giaccia A.J., Cochran J.R. (2014). An engineered Axl ‘decoy receptor’ effectively silences the Gas6-Axl signaling axis. Nat. Chem. Biol..

[32] Di Stasi R., Diana D., Capasso D., Palumbo R., Romanelli A., Pedone C., Fattorusso R., D’Andrea L.D. (2010). VEGFR1(D2) in drug discovery: Expression and molecular characterization. Biopolymers.

[33] Di Stasi R., Diana D., De Rosa L., Fattorusso R., D’Andrea L.D. (2019). Biochemical and Conformational Characterization of Recombinant VEGFR2 Domain 7. Mol. Biotechnol..

[34] Greenfield N.J. (2006). Using circular dichroism collected as a function of temperature to determine the thermodynamics of protein unfolding and binding interactions. Nat. Protoc..

